# Why do football clubs fail financially? A financial distress prediction model for European professional football industry

**DOI:** 10.1371/journal.pone.0225989

**Published:** 2019-12-26

**Authors:** David Alaminos, Manuel Ángel Fernández

**Affiliations:** 1 Department of Finance and Accounting, Universidad de Málaga, Málaga, Spain; 2 PhD Program in Mechanical Engineering and Energy Efficiency, Universidad de Málaga, Málaga, Spain; The Bucharest University of Economic Studies, ROMANIA

## Abstract

The study of financial distress has been the focus of financial research in recent decades and has led to the development of models for predicting financial distress that help assess the financial situation and the risks faced by companies. These models have focused exclusively on industrial and financial companies. However, a specific model that reflects the special characteristics of the football industry has not yet been created. Since recently the governing bodies of the football industry have increased the financial control of the clubs, as in the case of UEFA with the approval of the Financial Fair Play Regulation and demand a pronouncement on going concern in the annual financial statements of clubs as well as presenting a break-even deficit caused by losses, it seems necessary to have a model adapted to the characteristics of this industry. The present study provides a new model of prediction of financial distress for the football industry with an accuracy that exceeds 90%. It also offers a vision of the challenges facing the football industry in financial matters, helping the different interest groups to assess the financial solvency expectations of the clubs.

## Introduction

Insolvency is a global problem that affects companies of any country and industry, where the importance of control has increased in the last decade as a result of the financial crisis of 2008. This has evidenced the lack of effective and specific models that control the risk of insolvency and its prediction [1]. This is not an alien problem in the management of football clubs.

Recently, many European football clubs have shown financial problems derived from situations of great losses obtained in their activity. Only in the 2016/2017 period, the Union of European Football Associations (UEFA) investigated more than 70 clubs for this reason [2]. A situation of losses has repercussions of sports competitiveness since it makes impossible the improvement of the level of the players' staff, as well as it hinders the investments in the infrastructures of the club [3,4]. They also have administrative implications, and clubs are subject to economic sanctions and relegations [5]. Therefore, previous literature has pointed out that the balance of the club's profit and loss account is the variable that best explains the financial position of European club football [6–9]. In response to the serious financial situation of many clubs that regularly participate in European competitions, UEFA developed the concept of Financial Fair Play (FFP) as an extension of their licensing regulations [10]. The aim of this concept is to reduce the unfair financial game in European football (the non-payment of debts to other clubs or employees) and 'financial doping' (excessive financing to cover the losses derived from the expenses in player signings), putting the focus on the control of the financial statements of the clubs.

Some authors have conducted research on the insolvency of football clubs, but only developing broad proposals in terms of objectives and using variables taken from the general literature on insolvency [11–16]. However, following [17], by focusing the study on insolvency in a single industrial sector, more reliable databases are obtained, with a greater amount of personalized information, and therefore, the models created are more precise. On the other hand, existing models on insolvency prediction for the football industry have been designed to predict the bankruptcy of clubs, which was the most important concern in past decades. However, with the FFP in 2011, concerns about the financial situation of the clubs are focused on the losses generated, and not so much on bankruptcies, which are currently scarce. However, there are no models designed to predict future situations of insolvency from these situations, and the literature demands new models for predicting financial distress in order to meet the financial and legal needs raised in recent years [11,18].

In order to cover this gap, the present study develops a new financial distress prediction model for football clubs that responds to the most current concerns of the financial situation of the industry. To this end, a sample of European football clubs has been used, which includes clubs in a financial distress situation, defined in accordance with obtaining continuous losses, and clubs in non-financial distress. From this sample, data related to both the situation and the financial situation for the 2011–2016 period have been obtained, and logistic regression and neural network techniques have been applied. These methodologies have obtained the best precision results in previous literature on insolvency prediction [19]. In this way, useful conclusions have been obtained that the administrative bodies of football and any interest group in the industry can improve the financial control of the clubs.

The present study is organized as follows: Section 2 carries out a review of the literature on insolvency work in the football industry. Section 3 summarizes the scope and objectives of the FFP regulation as well as the financial control procedure. In section 4 the methods used are presented. In section 5 the data and the variables used in the research are detailed, and in section 6 the results obtained are analyzed. Finally, the conclusions of the study and its implications are exposed.

## Literature review

Starting from Beaver's seminal study on insolvency prediction [20], a large amount of literature has been created in this regard. [21] carried out the first important prediction research through the selection of accounting ratios, constructing a model called Z-Score. This model achieved to predict with 95% accuracy using the MDA method. [22] estimated the probability of insolvency with a Probit model, demonstrating that this probability decreases according to the return on assets, but which increases with respect to leverage. In the subsequent study of [23], the concept of insolvency is delimited, and an attempt is made to shorten the distance between the terms of financial distress and bankruptcy. Thus, while "bankruptcy" includes companies in a situation of legal insolvency, "financial distress" classify companies according to solvency ratios established by a reference criterion. Since then, most of the studies on financial distress prediction have formed databases of non-financial companies [24,25,17], industrial firms [26–28], and, in some cases, samples focused on specific industries, such as restaurants [29]. Most of these studies have highlighted the superiority of computational techniques in the construction of prediction models, and the good fit obtained with statistical methods [19].

For the football industry, the insolvency prediction studies have been referred to six countries, five of them were European (United Kingdom, Spain, Germany, Italy, and France) and one Asian (Turkey). Some of these studies have focused on the bankruptcy of clubs while others have analyzed various situations of payment failure, even though none of them has built a financial distress prediction model for the industry.

For English football, the study of [30] analyzed a sample of insolvent English clubs and concluded that managers' lack of planning to overcome low-income situations was the main cause of insolvency. Likewise, [31] focused the study of the insolvency for the English clubs in the cases of big expenses to improve the position of the club, and in the problems of productivity or demand. Their findings indicated that low productivity is the main insolvency factor, supporting the conclusions of [12]. [32] examined the history of financial instability and sports stability, in the sense of survival of the club, which is characteristic of English football and possibly much of world football. The document suggests that, although shareholders often lose money, clubs rarely disappear. It also suggests that while clubs are not immune to economic cycles, the impact is likely to be limited. [33] used a selection of financial and sports indicators, which are weighted according to their perceived relative importance and in relation to the components of financial management and regulations of the governing bodies. The study used data belonging to Premier League clubs, where the financial turnover ratio and the win ratio are shown as the most significant indicators. [34] analyzed the payments made by the Premier League to the relegated clubs in order to mitigate the negative impact of the decline. Their results showed that an increase in the number of clubs with these grants and their average value coincides with a reduction in the competitive balance of the championship. In addition, the clubs that receive these grants are twice as likely to ascend to the English Premier League and are less likely to descend to the third category.

For the Spanish football industry, [35] indicated that the great cultural and historical importance of the clubs causes great incentives for their financial rescue by local authorities. [27] also analyzed the factors that allow clubs to overcome situations of insolvency. Their results indicate that the most significant factors are a lower ratio of tangible assets, adjustments in wages, and high leverage. This expands the set of indicators that show the financial distress in Spanish clubs, where [36] argued that an increase in salary spending would cause financial distress. In the same line, [37] added the importance of the club’s division and the revenues. On the other hand, [38] studied the explanatory variables in the cases of bankruptcy of Spanish clubs using logistic regression techniques. Their conclusions indicated that sport performance variables were significant, but financial variables had no impact on the problem studied. However, [11] used the Z-Score ratios and pointed out the importance of increasing revenues and reducing wages to achieve the clubs’ financial stability.

Regarding German football, [39] showed how German regulation affects the governance structure of clubs by excessively dividing the voting power without considering the proportion of ownership and interests of each shareholder. Likewise, the low accounting standards of clubs invite to hide insolvency situations. [15] showed how the increase in the total debt of the German league came from parts of the two main clubs (Bayern Munich and Borussia Dortmund). [40] analyzed the overinvestment produced in the German league by hiring players. Their results indicate that aspects of German regulation, such as restricting new shareholders from taking the power of the club and the introduction of the FFP, have not been able to solve moral hazard problems in insolvency management. [41] showed that the clubs go into insolvency when the team sport performance has been much worse than expected, and this situation causes lower revenues.

For the Italian clubs, [14] showed that the causes of financial problems were different depending on the size of the club. For bigger clubs, the main cause was profitability, and for smaller clubs, bad investment decisions. The same conclusions were obtained by [42], also pointing out that the fundamental items for the financial control of the clubs are salaries and income from the sale of players. [43] examined the relationship between human capital investments and financial performance. They argue that the presence of the family board has a moderating effect on the spend decision.

For the French clubs, [16,44] indicated that French football is characterized by lax financial management and a soft budget constraint and that this causes the creation of weak governance structures, in which shareholders behave as non-profit investors profit. His recommendations included strengthening the governance structure and restoring financial discipline. [45] found that abrupt changes in demand and a property structure with professional status are related to payment failure, and [46] that more revenue affects sporting achievements and these, in turn, positively impact the financial situation. Finally, for Turkish football, [47,48] noted that leverage is the most significant variable in the club’s financial performance.

The regulation of FFP has been shown with the sole objective of preventing the entry of large benefactors (as owners) that introduce money not produced in the activity of the football industry [49–51]. [9] argued that with greater regulation, such as the FFP, it can increase the imbalance in the leagues because it can protect well-established clubs against emerging clubs. [52] supported these conclusions after analyzing and comparing the regulation of English and German football. [53] has shown that the FFP has been able to improve financial recovery but has been able to increase polarization. Recent empirical works such as [54,55] also concluded that the FFP rules have been able to improve the financial recovery of clubs but have been able to increase polarization and imbalances within the leagues.

## Scope and aims of Financial Fair Play

### Overview

The FFP regulation [5] applies to clubs participating in competitions organized by UEFA. This regulation organizes the rights, obligations, and commitments of all the agents interested in the club licensing system and defining the minimum requirements that a UEFA member federation must fulfill to serve as a licensor of the clubs in its respective country. It also structures the basic actions that the licensor must apply in reference to minimum criteria on sports, infrastructure, administrative, legal and financial issues.

In addition, it defines the actions and work of the UEFA Financial Club Control Body, the basic actions that the licensors must comply within their analysis of the conditions of control of the club, the obligations of the licensees with respect to the competitions they play, and the monitoring tasks that the holders of these licenses must fulfill.

The regulation of the FFP marks as one of its main objectives to achieve a financial "fair play" in club competitions under the UEFA system. With this objective, we try to increase the economic and financial capacity of clubs, improve their transparency and credibility, prioritize the protection of creditors and ensure that clubs do not comment on defaults with respect to the salaries of their players, their fiscal responsibilities and debts with other clubs. It is intended that the financial area of football clubs gain in discipline and rationality, encouraging them to operate on the basis of their own income, and abandoning other sources of funding incompatible with European regulations [56].

### Control procedure on financial viability

With regard to the annual financial information that must be submitted by the clubs competing under the UEFA system, the FFP requires that the annual financial statements must be audited by an independent auditor, and their annual accounts being structured in the following structure [5]: a) the balance sheet; b) the profit and loss account; c) the statement of cash flow; d) a summary of significant accounting policies and other actions; e) a financial review by club managers. In addition, and in the event of any of the indicators defined below being violated, the license applicant must present future financial information to demonstrate to the licensor its ability to continue with its usual activity until the end of the season. In other words, a license applicant club that shows negatively the conditions defined by the following indicators will be considered as infringing:

1. Indicator 1: Going concern. The auditor's report regarding the annual financial statements must decide on going concern.
2. Indicator 2: Negative equity. A position of equity that has deteriorated significantly compared to the figure calculated in the annual financial statements of the previous year.
3. Indicator 3: Break-even result. A break-even deficit caused by losses.
4. Indicator 4: Sustainable debt indicator. The debt is greater than EUR 30 million and it is greater than 7 times the average of the earnings.
5. Indicator 5: Sustainable debt indicator. The debt is greater than EUR 30 million and it is greater than 7 times the average of the earnings
6. Indicator 6: Player transfer balance. A player transfer deficit greater than EUR 100 million.

## Methodology

### Logistic regression

This study uses a Logit model in order to predict financial distress based on observable variables of the football clubs. The model specification may be represented via Eq (1).

$$y_{i}^{*}=x_{i}+\varepsilon_{i}$$ (1)

With

$$y_{i}=\left\{ \begin{array}{l} 1\;if\;{\text{y}}_{i}^{*}>0.5\\ 0\;if\;{\text{y}}_{i}^{*}\leq 0.5\; \end{array}\right.$$

Then

$$P[y_{i}=1]=P[x_{i}\beta +\varepsilon_{i}>0.5]=F(x_{i}\beta )$$ (2)

$$P[y_{i}=0]=1-F(x_{i}\beta )$$ (3)

As [57] described, models with dependent discreet variables frequently appear as index function models; that is, it interprets the result of a discrete choice as a reflection of an underlying regression. In the case of this study, the dependent variable (*y*_*i*_^*m*^) has a value of 1 if the company is bankrupt and has a value of 0 otherwise. In this study is identified a football club as financial distress in accordance with the criterion used by [1,58,59]: (1) Negative EBITDA interest coverage; (2) Negative EBIT; and (3) Negative net income before special items. The parameters vector *β* reflects the impact that the independent variables vector *x*_*i*_ has on the probability of financial distress. The logistic distribution function is applied, and the model was calculated using the backward steps method, where the removal of variables is based on the probability of the plausibility statistic, which, in turn, is based on estimates of maximum partial plausibility. If the probability estimated is greater than 0.5, the prediction is that it does belong to the group of football clubs in financial distress, otherwise it is assumed to belong to the other group considered. As such, from Eq (1):

$$P(y_{i}=1)=\frac{e^{\beta \prime x}}{1+e^{\beta \prime x}}=\frac{1}{1+e^{-(\beta \prime x)}}$$ (4)

Thus, the ratio between the two probabilities (known as the Odds ratio) is established as follows in Eq (5).

$$Odds=\frac{P(y_{i}=1)}{1-P(y_{i}=1)}=\frac{1/[1+e^{-(\beta \prime x)}]}{1/[1+e^{\left(\beta \prime x\right)}]}=\frac{1+e^{\left(\beta \prime x\right)}}{1+e^{-(\beta \prime x)}}=e^{\left(\beta \prime x\right)}$$ (5)

The estimated coefficients (β) represent measurements of the changes in the odds ratio. In this regard, a positive coefficient increases the probability of the event occurring, whilst a negative value decreases the probability of its occurrence [60]. The Odds ratios may be interpreted as the number of times that the phenomenon is more likely to occur than that it is not.

By applying the logarithms in Eq (5) we obtain Eq (6), which is a linear expression of the model under consideration.

$$y_{i}{}^{*}=\text{ln}\frac{P(y_{i}=1)}{1-P(y_{i}=1)}=\text{ln}e^{\beta \prime x}=\beta \prime x$$ (6)

### Multilayer perceptron

**Fig 1:**
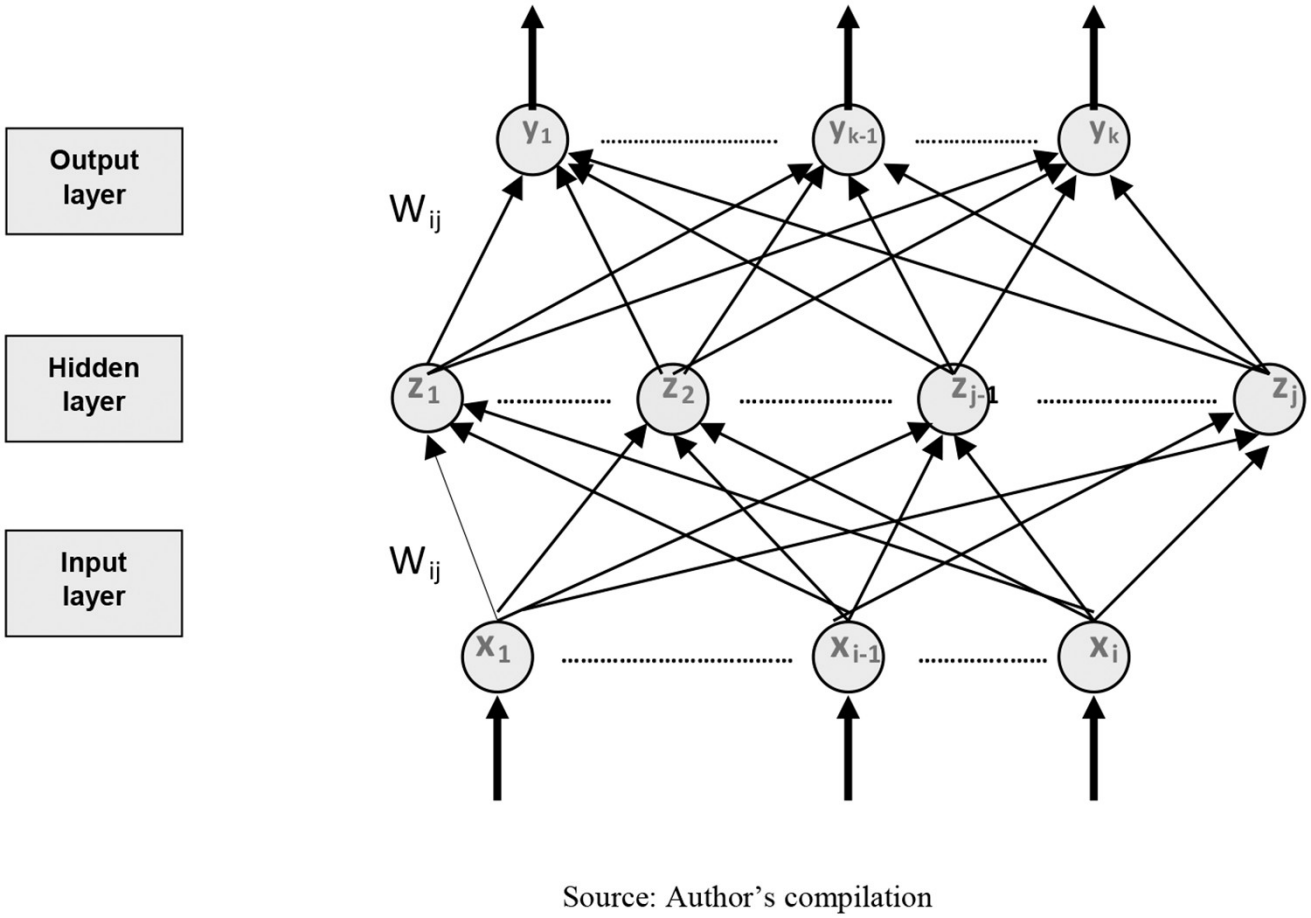
MLP architecture.

A Multilayer Perceptron (MLP) is a feedforward artificial neural network model of supervised learning, which is composed of a layer of input units (sensors), another output layer and a certain number of intermediate layers, called hidden layers in so much that they do not have connections with the outside. Each input sensor would be connected to the units of the second layer, these in turn to those of the third layer, and so on (Fig 1). The network will aim to establish a connection between a set of input data and a set of desired outputs.

[61] confirmed that the learning in MLP represented a special case of a functional approach, where there is no assumption about the underlying model of the data analyzed. This process involves finding a function that correctly represents learning patterns, as well as carrying out a process of generalization that allows efficiently treating individuals not analyzed during said learning [62]. This is done by adjusting weights *W* from the information coming from the sample set, considering that both the architecture and the connections of the network are known, being the objective to obtain those weights that minimize the learning error. Given, then, a set of pairs of learning patterns {(x_1_, y_1_), (x_2_, y_2_) … (x_p_, y_p_)} and an error function ε (W, X, Y), the training process involves the search for the set of weights that minimizes the learning error E (*W*) [63], as expressed in Eq (7).

$$min_{W}E\left(W\right)=min_{W}\sum_{i=1}^{p}\varepsilon (W,x_{i},y_{i})$$ (7)

Most of the analytical models used to minimize the error function use methods that require the evaluation of the local gradient of the E (*W*) function and techniques based on second order derivatives can also be considered [62]. In addition, and in order for MLP to report the importance of each variable in the results of the constructed model, it is possible to perform a sensitivity analysis [63,64]. This analysis consists of taking 100% of the data and dividing them into groups, and each group of data is processed in the network built as many times as there are model variables. Each time the value of one of the variables is modified, placing it with zero value. The responses of the network are evaluated in relation to the objective values or classification values already known, by means of Eq (8).

$$Sx_{i}=\sum_{j=1}^{n}(\Phi x_{ij}(o)-\Phi x_{ij}{)}^{2}$$ (8)

where *Φx*_*ij*_ (0) is the value of the output of the network when the variable *X*_*ij*_ is zero, *Φx*_*ij*_ is the classification value already known, *X*_*i*_ is the variable whose importance is to be established, and *S*_*xi*_ is the sensitivity value of the variable.

## Sample, data and variables

The present research uses a sample of 234 European professional football clubs that have participated during the period 2013–2016 (S1 Table). This sample consists of 93 clubs with financial distress (FD = 1) and the rest clubs without financial distress (FD = 0). Given the importance that the generation of profits has for the solvency of European football clubs (UEFA, 2018), it has been used as a proxy for financial distress three demands of negative results for two consecutive years [1,58,59]: (1) Negative net income before special items; (2) Negative EBIT; and (3) negative EBITDA interest coverage. The sample is divided into three sub-samples according to the time horizon prior to the situation of financial distress: one year (t-1), two years (t-2) and three years (t-3) prior to the financial distress status (S1–S3 Files).

The financial information of the clubs in the sample has been obtained from the Amadeus database of Bureau Van Dijk (see: https://amadeus.bvdinfo.com/), which provides economic and financial data of more than 24 million European companies. As of NACE Code 2 -activities of sports clubs (code 9312)- all those companies that have as main activity the practice of football as a business have been chosen. The specific information about the football industry has been extracted from the Transfermarkt web portal (see: https://www.transfermarkt.com/). The corporate governance variables have been selected from the experience of [43]. The performance variables have been extracted from previous cases [11,33,38]. Finally, the financial variables have been chosen from the previous works of [11,34,40,42,45–48]. Table 1 shows the definition of the explanatory variables used.

**Table 1:** Explanatory variables definition.

Attribute	Code	Variables	Expected Sign
**Corporate Governance Factors**	I1	Institutional Ownership (dummy)	+
	I2	N° of Shareholders	-
	I3	N° of Members of the Board of Directors	-
	I4	CEO Duality (dummy)	+
**Performance Factors**	P1	Main Club of the City (dummy)	-
	P2	Population of the City (Club Market)	-
	P3	Average Attendance at the Stadium	-
	P4	Accumulated points	-
	P5	Promotion /Relegation	-
	P6	Division	+
	P7	Performance Ratio (Szymanski Ranking^1^)	-
	P8	Wage Bill^2^	-
**Financial Factors**	F1	Current Liabilities/Current Assets	+
	F2	Total Debt/Total Assets	+
	F3	Total Debt/Total Revenue	+
	F4	Expenses on Players/Operating Revenue	+
	F5	Working Capital/Total Assets	-
	F6	Retained Earnings/Total Assets	-
	F7	EBIT/Total Assets	-
	F8	Sales/Total Assets	-
	F9	Total Liabilities/Total Assets	+
	F10	Total Liabilities/Equity	+
	F11	Short-term Liabilities/Equity	+
	F12	Fixed Assets/Equity	+/-
	F13	Net Profit/Number of Shares	-
	F14	Net Capital/Equity	-
	F15	EBIT/Sales	-
	F16	Net Income Growth	-
	F17	Net Sale Growth	-
	F18	Asset Growth	+/-
	F19	Liabilities Growth	+
	F20	Debt Coverage Ratio	-

^1^ Szymanski Ranking = -ln(p/43-p). The total of clubs that participate in First and Second Division is 42, where one more must be added counting back to the given club with which it is working. The term "p" represents the final position that each club reached at the end of the season.

^2^ Wage Bill is measured by the salary expenses of the players of a given club divided by the total of the National League (expressed in millions of Euros).

In addition, in order to test the reliability and predictive capability of the models, different test samples were used, and unrelated to those used in estimating the models. From a random selection, it reserved 70% of the data to build training samples and the remaining 30% to obtain testing samples. In the case of MLP, 500 sets of variables were randomly selected, to which 10-fold cross-validation was applied.

## Results

For the analysis of the results in the present work, a descriptive study of the variables used is presented first. Subsequently, the development of the prediction model applying the proposed methods.

### Descriptive statistics

The main descriptive statistics of the variables that make up the sample appear in S2 and S3 Tables. Regarding corporate governance variables, note that football clubs that are not in financial distress have twice as many shareholders (8) than football clubs that are in financial distress (4). It is also noted that football clubs that are in financial distress have an average incidence of being the main club of their cities of 0.452, lower than football clubs that are not in financial distress, whose value is 0.697. On the other hand, the variable Promotion / Relegation (P5) shows that clubs that descend category are more prone to financial distress. In addition, the variable of Wage Bill (P8) shows a higher average expenditure in salaries in football clubs that are in financial distress, different from the previous literature [26].

Likewise, the football clubs sample shows a high level of leverage during the study period, with average values of Total Liabilities / Total Assets (F9) which in all cases exceeds 90%. Finally, and in terms of return on assets (F7), the clubs have average values that range between 11.0% and 12.80% in cases of non-financially distressed clubs, and between -9.30% and -15.70% in cases of financially distressed clubs.

In addition, there is also a moderate dispersion in the distribution of the variables analyzed with respect to the values of their means, which is extendable to the entire sample.

### Classification results

Tables 2 and 3 show the results obtained with the different methods proposed. With Logit, the classification level in the training sample amounts to 85.62%, 78.36%, and 81.94%, for t-1, t-2, and t-3, respectively. In the testing sample, the classification level also remains close to 80%, specifically, 81.35%, 77.64%, and 74.87%. Likewise, the ROC curve corresponding to these Logit models indicates a correct classification in all cases (0.907, 0.875, 0.834) (S1 and S2 Figs). On the other hand, using MLP, the classification level in the training sample amounts to 95.92%, 90.39, and 86.95%, and in the testing sample, to 93.89%, 86.33%, and 81.35%, for t-1, t-2 and t-3, respectively. In addition, for the MLP models obtained, the ROC curve also indicates a correct classification (0.960, 0.842 and 0.795), but only exceeds Logit in the model built with information corresponding to t-1. In this way, we can see that in all cases the level of accuracy of MLP is greater than that of Logit, both in the simple training and in the simple testing. However, using Logit we have obtained more robust models when we use information far from the time horizon of financial distress, specifically for t-2 and t-3.

**Table 2:** Results of the estimated Logit models.

Specification Models		Summary				Classification Accuracy (%)	
		Omnibus Test	Hosmer-L. Test	ROC Curve	R^2^ Nagelk.	Training Sample	Testing Sample
t-1	*Y*_t-1_ = 0.713+1.293I1** − 0.420I3-1.754P1***-1.580P5+0.293F1***-0.437F3**-1.532F20***	0.000***	0.725***	0.907	0.456	85.62	81.35
t-2	*Y*_t-2_ = -0.104–0.027I2*** -1.144P1*** +10.095F1**-9.789F3*** +180.244F5***-80.012F8***-44.671F14***	0.004***	0.792***	0.875	0.367	78.36	77.64
t-3	*Y*_t-3_ = 14.319–2.547P1** +0.081P3**-4.705P5***-3.423P8+-9.920F2*** +15.182F3**-19.192F5***-3.799F6***-12.419F7**	0.004***	0.882***	0.834	0.558	81.94	74.87

**Sig. at 0.05

***Sig. at 0.01

**Table 3:** Results of the estimated MLP models.

Sample	Classification (%)		RMSE		ROC Curve	Significant Variables
	Training	Testing	Training	Testing		
t-1	95.92	93.89	1.36	1.52	0.960	P4, F3, F8, F11, F19, F20
t-2	90.39	86.33	1.67	1.83	0.842	F1, F2, F3, F5, F11, F15, F16, F20
t-3	86.95	81.35	1.86	2.13	0.795	I1, P3, F2, F3, F4, F5, F9, F12

RMSE: Root mean squared error; Significant variables: Normalized important > 50%

As indicated above, the results obtained indicate a set of significant variables that are repeated in practically all the methodologies used. One of these variables is the main club in the city (P1), which has been significant in the three Logit models, being the main club of the city related to a higher level of sales and attendance at the stadium, and closely linked to the economic profitability of the club [45]. On the other hand, the variable Total Debt / Total Revenue (F3) has also been a recurring ratio, reflecting the importance of the proportion of the debt over the income obtained by the club, very related to the ability to pay the debt as with the margin to make investments, such as in new signings of players [38,46]. Finally, the variable Current Liabilities / Current Assets (F1) has also been significant in the Logit models for one and two years prior to the state of financial distress, which highlights the great importance of liquidity for the company's financial viability, just as it has had been contrasted in different previous studies [47,38,46]. Therefore, according to the results of Logit, clubs that are not main clubs in their cities, which have high debt and low liquidity are more likely to enter into financial distress.

Regarding MLP results, the Total Debt / Total Revenue variable (F3) reappears as one of the most repeated, appearing in the three temporal models. On the other hand, there is a group of variables that appear in at least two models of MLP, such as Total Debt / Total Assets (F2), which shows the level of indebtedness of the club with respect to the amount of assets, appearing in models of greater temporal horizon, variable that has not been significant in previous studies. Other debt variables appear as the most repeated, such as Short-Term Liabilities / Equity (F11) and Debt Coverage Ratio (F20), which show the importance of the club's ability to pay short-term debts, as well as previous jobs such as [48]. Finally, there are also liquidity variables such as Working Capital / Total Assets (F5), which details the proportion of liquid left by the clubs with respect to total assets, appearing in the previous literature as significant [11]. Therefore, according to the results with MLP, clubs with a high level of indebtedness and low liquidity ratio are more likely to be in a state of financial distress.

These results for the football industry are different from those obtained for other sectors such as the industrial or financial sector. In the first place, due to the variables of the football industry that have been significant in the developed model and that, logically, do not appear in the previous works on other sectors. Such is the case of the ratio of the main club in the city. Second, only one variable has been common with the general models and referred to Total Debt / Total Revenue (F3). This variable of indebtedness is significant in many previous models [1,57]. In third place, we have also been able to verify that certain variables that have been significant in previous generic works are not for the football industry. For example, profitability and activity have been outstanding predictors of financial distress for the manufacturing, commercial and service sectors [58,65–67]. In fourth place, other specific variables to the football industry, such as Division (P6) and Performance ratio (P7), have not been significant in the present study, although in other previous works [38,45]. These two variables refer to sports performance taking into account the relative position of the club with respect to the other professional clubs that have competed in both the first and second division. The difference in results may be due to the fact that the previous works used information corresponding to periods prior to the financial requirements of the FFP, or because their results were limited to only certain countries. For example, [45] conducted their study with information from the period 1970–2014 and for the French competition. Therefore, the significant variables in the prediction model of financial distress for the football industry form a unique set and different from what has been appropriate for other sectors. Our results show how significant ratios related to profitability and indebtedness, and this may be due to income imbalances advocated by previous work on the consequences of the FFP [51,55]. But it also shows how it is necessary to improve these financial rules to improve the financial situation of clubs by reducing their expectations of financial distress [50,51].

Finally, the results obtained in this study also show that MLP achieves greater accuracy than Logit. MLP models achieve 93%, 86% and 81%, for t-1, t-2 and t-3, respectively, while for Logit, models achieve 81%, 77% and 74%. This evidences that computational techniques, and especially MLP, show more accurate results in insolvency prediction studies [19,68].

## Conclusions

The principle of financial distress is one of the most important in the solvency of a company. Because of this, it has been the focus of financial research for decades, which has developed models to assess financial distress of industrial and financial companies. However, scarce models have been developed exclusively for the football industry.

The activity of football clubs is unique in many ways, and therefore, it seems important that both financial managers and other interest groups as investors have tools specially designed for their management. Currently, one of the concerns of the industry is the financial situation of clubs, and therefore the literature demands new models of financial distress prediction that explain the causes of a situation of insolvency. In order to respond to this demand, the present study builds a new prediction model adapted to the current financial distress of football clubs. This new model can constitute an empirical instrument of control over the indicators referred to in the regulation of the FFP, and in the same way, it can improve the recommendations to the clubs that present financial reports that do not comply with said indicators by allowing to quantify the degree of financial distress.

The model developed in this study has shown that low liquidity, high leverage, poor sports performance and small size of the club market are the best predictors of the distress of football clubs. In addition, with MLP methodology, it is possible to obtain high accuracy rates in the prediction of financial distress (higher than 90% in t-1).

The importance of the football industry in Europe, which represents a relevant pillar in the social-economic dynamism, suggests greater involvement of the research tasks. Addressing this gap from a scientific point of view, trying to improve the set of tests that allow a better prediction and analysis of the clubs leads to a research milestone from which future lines of research can be derived. Thus, it would be interesting to contrast the results of this paper for models developed with samples from clubs belonging to other regions of the world, for example, the South American Football Confederation (CONMEBOL) and the Confederation of North America, Central America and the Caribbean Football (CONCACAF), which would allow knowing the moderating effect of other cultural and legislative environments on the viability of the clubs. Also, check the predictive capacity of our model to predict financial distress in other sports industries, because it takes into account performance characteristics of the football industry and these could be extrapolated to clubs of different disciplines.

## Supporting information

S1 Table Observations by National League. (DOCX)

S2 Table Descriptive statistics. (DOCX)

S3 Table Descriptive statistics. (DOCX)

S1 Fig ROC curves of estimated Logit models. (TIF)

S2 Fig ROC curves of estimated MLP models. (TIF)

S1 File Sample t-1. Variables of financially distressed /non-financially distressed European football clubs in t-1.
(XLSX)

S2 File Sample t-2. Variables of financially distressed /non-financially distressed European football clubs in t-2.
(XLSX)

S3 File Sample t-3. Variables of financially distressed /non-financially distressed European football clubs in t-3.
(XLSX)
