# Supplementary material for: Why do football clubs fail financially? A financial distress prediction model for European professional football industry
Source: PLoS One. 2019 Dec 26;14(12):e0225989. doi: 10.1371/journal.pone.0225989 (PMC6932787; doi:10.1371/journal.pone.0225989)
Supplement: S1 Table — (DOCX) [file pone.0225989.s001.docx]

**S1 Table. Observations by National League**

| **National League** | **FD=1** | **FD=0** |
| --- | --- | --- |
| Belgium | 4 | 8 |
| Bulgaria | 1 | 2 |
| Croatia | 0 | 2 |
| Cyprus | 0 | 1 |
| Czech Republic | 0 | 2 |
| Denmark | 4 | 3 |
| England | 26 | 15 |
| France | 13 | 9 |
| Germany | 4 | 25 |
| Greece | 1 | 4 |
| Italy | 17 | 10 |
| Netherlands | 5 | 7 |
| Norway | 0 | 3 |
| Poland | 2 | 3 |
| Portugal | 1 | 6 |
| Romania | 0 | 2 |
| Russia | 1 | 0 |
| Scotland | 2 | 4 |
| Spain | 9 | 28 |
| Sweden | 0 | 2 |
| Switzerland | 0 | 1 |
| Turkey | 1 | 1 |
| Ukraine | 2 | 2 |
