# Supplementary material for: Why do football clubs fail financially? A financial distress prediction model for European professional football industry
Source: PLoS One. 2019 Dec 26;14(12):e0225989. doi: 10.1371/journal.pone.0225989 (PMC6932787; doi:10.1371/journal.pone.0225989)
Supplement: S2 Table — (DOCX) [file pone.0225989.s002.docx]

**S2 Table. Descriptive statistics**

|  |  |  | **I1** | **I2** | **I3** | **I4** | **P1** | **P2** | **P3** | **P4** | **P5** | **P6** | **P7** | **P8** |
| --- | --- | --- | --- | --- | --- | --- | --- | --- | --- | --- | --- | --- | --- | --- |
| **(t-1)** | **FD= 1** | Mean | 0.495 | 4.734 | 19.454 | 0.627 | 0.452 | 1035557.306 | 19015.909 | 53.630 | -0.045 | 1.641 | 1.329 | 193.003 |
|  |  | SD | 0.503 | 8.482 | 11.237 | 0.416 | 0.526 | 2845.227 | 131.382 | 15.844 | 0.453 | 0.779 | 0.970 | 15.922 |
|  | **FD= 0** | Mean | 0.338 | 8.284 | 17.578 | 0.372 | 0.697 | 819346.086 | 23771.694 | 56.099 | 0.038 | 1.356 | 1.699 | 174.131 |
|  |  | SD | 0.475 | 9.877 | 12.128 | 0.733 | 0.461 | 1911.81 | 185.539 | 15.837 | 0.417 | 0.568 | 0.951 | 14.115 |
| **(t-2)** | **FD= 1** | Mean | 0.491 | 4.871 | 20.824 | 0.517 | 0.449 | 1032487.381 | 18868.325 | 52.973 | -0.055 | 1.576 | 1.614 | 208.855 |
|  |  | SD | 0.507 | 8.456 | 11.417 | 0.247 | 0.517 | 2617.096 | 131.123 | 14.913 | 0.376 | 0.745 | 0.766 | 17.622 |
|  | **FD= 0** | Mean | 0.335 | 8.173 | 17.923 | 0.483 | 0.656 | 817835.172 | 24144.455 | 54.949 | 0.038 | 1.379 | 1.785 | 174.797 |
|  |  | SD | 0.472 | 9.428 | 12.224 | 0.372 | 0.427 | 1911.81 | 192.824 | 16.079 | 0.417 | 0.612 | 0.837 | 13.432 |
| **(t-3)** | **FD= 1** | Mean | 0.497 | 4.246 | 20.156 | 0.518 | 0.452 | 1027014.508 | 19188.256 | 53.662 | -0.008 | 1.565 | 1.374 | 125.648 |
|  |  | SD | 0.509 | 8.174 | 11.887 | 0.562 | 0.505 | 2672.742 | 134.608 | 14.686 | 0.365 | 0.760 | 1.012 | 13.087 |
|  | **FD= 0** | Mean | 0.332 | 8.924 | 17.972 | 0.359 | 0.683 | 816201.736 | 24635.173 | 54.278 | 0.053 | 1.438 | 1.547 | 103.071 |
|  |  | SD | 0.473 | 7.382 | 12.375 | 0.482 | 0.491 | 1933.81 | 191.381 | 16.847 | 0.397 | 0.682 | 0.976 | 12.982 |
