# Supplementary material for: Why do football clubs fail financially? A financial distress prediction model for European professional football industry
Source: PLoS One. 2019 Dec 26;14(12):e0225989. doi: 10.1371/journal.pone.0225989 (PMC6932787; doi:10.1371/journal.pone.0225989)
Supplement: S3 Table — (DOCX) [file pone.0225989.s003.docx]

**S3 Table. Descriptive statistics**

|  | |  |  | **F1** | **F2** | **F3** | **F4** | **F5** | **F6** | **F7** | **F8** | **F9** | **F10** | **F11** | **F12** | **F13** | **F14** | **F15** | **F16** | **F17** | **F18** | **F19** | **F20** |
| --- | --- | --- | --- | --- | --- | --- | --- | --- | --- | --- | --- | --- | --- | --- | --- | --- | --- | --- | --- | --- | --- | --- | --- |
| **(t-1)** | **FD= 1** | | Mean | 3.747 | 0.789 | 2.604 | 1.478 | 0.521 | -0.344 | -0.157 | 0.551 | 0.928 | 12.503 | 14.571 | 1.390 | -1.264 | 10.110 | -0.161 | -0.157 | 0.372 | 0.104 | 0.211 | 0.131 |
|  |  |  | SD | 5.292 | 0.585 | 1.697 | 1.125 | 0.753 | 0.765 | 0.230 | 1.129 | 0.903 | 10.666 | 8.040 | 1.819 | 2.834 | 12.369 | 0.772 | 0.604 | 0.482 | 0.793 | 0.964 | 0.682 |
|  | **FD= 0** | | Mean | 2.230 | 0.576 | 1.768 | 0.934 | 0.477 | 0.158 | 0.110 | 0.919 | 0.957 | 19.112 | 12.172 | 7.524 | 2.837 | 16.423 | 0.137 | 0.128 | 0.502 | 0.127 | 0.007 | 0.170 |
|  |  |  | SD | 2.715 | 0.205 | 1.744 | 1.634 | 0.498 | 0.265 | 0.287 | 0.719 | 0.910 | 11.042 | 6.782 | 8.837 | 1.319 | 12.213 | 0.775 | 0.529 | 1.725 | 0.793 | 0.821 | 0.452 |
| **(t-2)** | **FD= 1** | | Mean | 3.388 | 0.691 | 2.448 | 1.503 | 0.413 | -0.678 | -0.111 | 0.846 | 0.921 | 12.492 | 13.709 | 1.451 | -4.375 | 10.773 | -0.148 | -0.154 | 0.140 | 0.173 | 0.177 | 0.194 |
|  |  |  | SD | 5.039 | 0.544 | 1.853 | 1.198 | 0.248 | 1.333 | 0.178 | 0.513 | 0.888 | 9.486 | 10.753 | 1.985 | 9.927 | 14.968 | 0.830 | 0.926 | 0.540 | 0.741 | 0.035 | 0.720 |
|  | **FD= 0** | | Mean | 2.495 | 0.528 | 1.389 | 0.884 | 0.404 | 0.180 | 0.105 | 0.927 | 0.918 | 11.743 | 11.106 | 8.182 | 2.941 | 13.213 | 0.144 | 0.162 | 0.608 | 0.117 | 0.101 | 0.147 |
|  |  |  | SD | 3.046 | 0.644 | 1.145 | 1.892 | 0.237 | 0.634 | 0.157 | 0.922 | 0.873 | 7.364 | 11.651 | 9.350 | 1.255 | 11.629 | 0.606 | 0.983 | 2.262 | 0.510 | 0.270 | 0.517 |
| **(t-3)** | **FD= 1** | | Mean | 2.333 | 0.610 | 1.994 | 1.603 | 0.431 | -0.875 | -0.093 | 0.930 | 0.947 | 18.602 | 12.803 | 2.540 | -2.761 | 16.944 | -0.123 | -0.148 | 1.124 | 0.167 | 0.171 | 0.104 |
|  |  |  | SD | 4.586 | 0.340 | 1.677 | 1.645 | 0.403 | 0.621 | 0.144 | 0.569 | 0.919 | 11.687 | 8.456 | 5.508 | 6.607 | 14.544 | 0.713 | 0.019 | 0.020 | 0.188 | 0.168 | 0.746 |
|  | **FD= 0** | | Mean | 2.718 | 0.572 | 1.130 | 0.898 | 0.418 | 0.119 | 0.098 | 0.951 | 0.929 | 12.802 | 10.345 | 9.596 | 2.198 | 13.043 | 0.138 | 0.147 | 1.674 | 0.120 | 0.153 | 0.153 |
|  |  |  | SD | 4.431 | 0.785 | 2.340 | 1.583 | 0.245 | 0.235 | 0.161 | 0.976 | 0.880 | 8.530 | 10.520 | 9.609 | 1.158 | 11.529 | 0.543 | 0.336 | 12.643 | 0.533 | 0.350 | 0.529 |
