# Supplementary figures and images for: Why do football clubs fail financially? A financial distress prediction model for European professional football industry
Source: PLoS One. 2019 Dec 26;14(12):e0225989. doi: 10.1371/journal.pone.0225989 (PMC6932787; doi:10.1371/journal.pone.0225989)

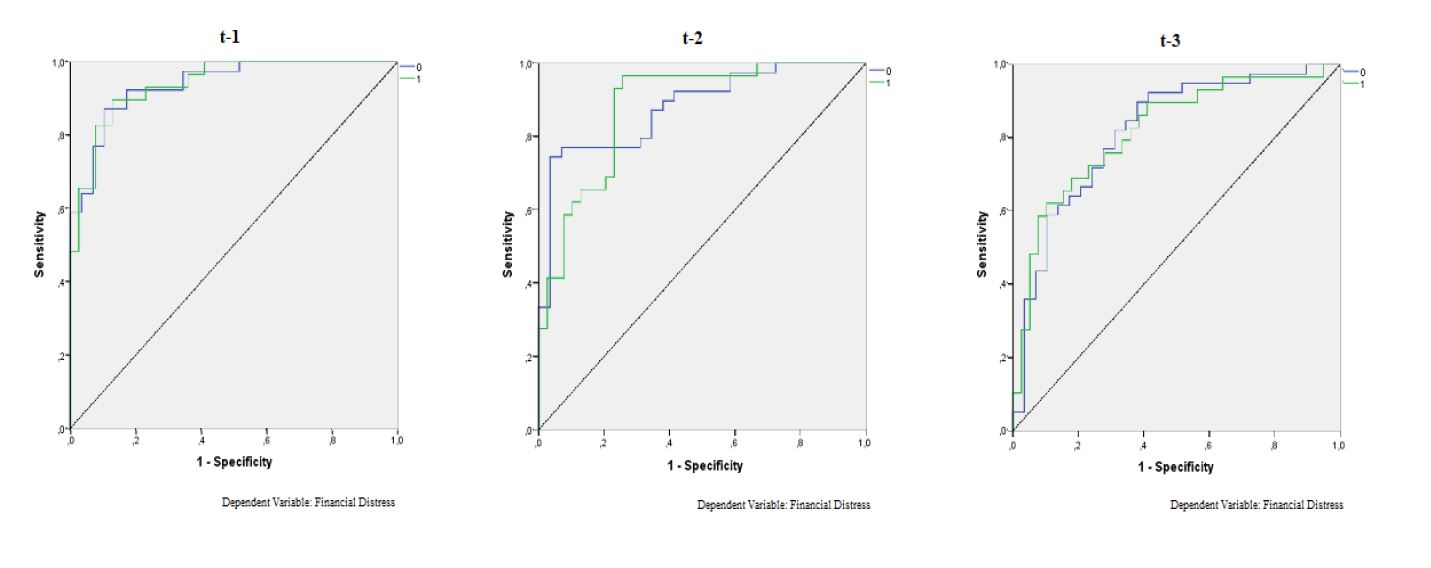

Supplement: S1 Fig — (TIF) [file pone.0225989.s004.tif]

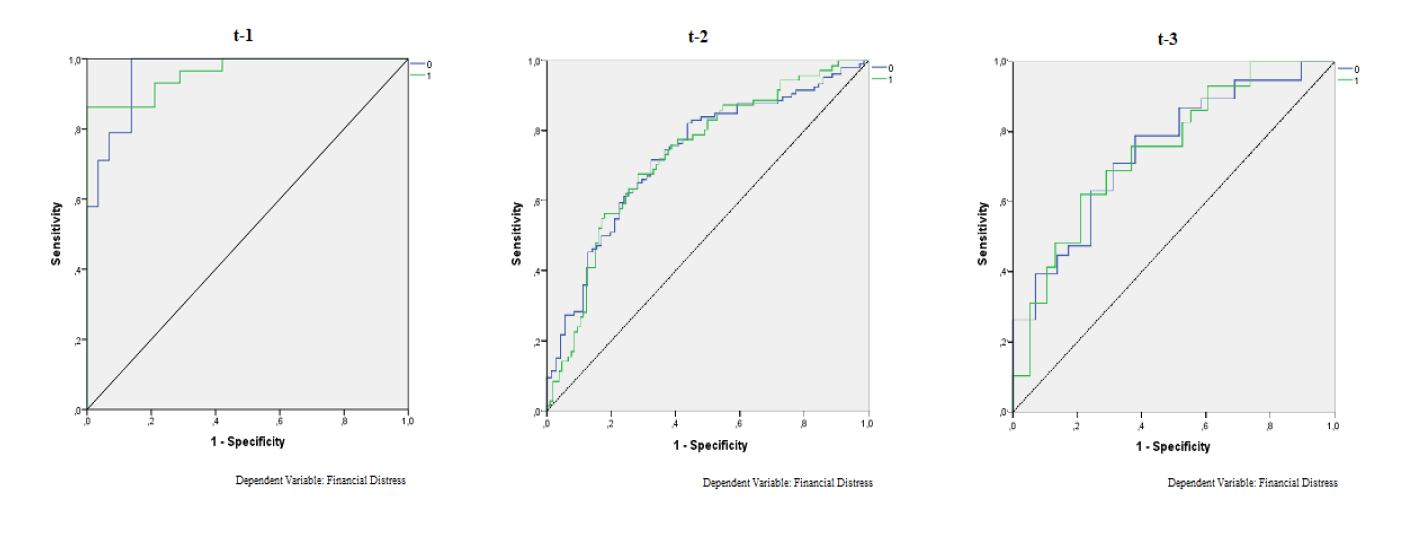

Supplement: S2 Fig — (TIF) [file pone.0225989.s005.tif]
